# Anti-Proliferative Effects of *Siegesbeckia orientalis* Ethanol Extract on Human Endometrial RL-95 Cancer Cells

**DOI:** 10.3390/molecules191219980

**Published:** 2014-12-01

**Authors:** Chi-Chang Chang, Hsia-Fen Hsu, Kuo-Hung Huang, Jing-Mei Wu, Shyh-Ming Kuo, Xue-Hua Ling, Jer-Yiing Houng

**Affiliations:** 1 Institute of Biotechnology and Chemical Engineering, I-Shou University, Kaohsiung 84001, Taiwan; E-Mails: ed101779@edah.org.tw (C.-C.C.); pt153575@yahoo.com.tw (K.-H.H.); ed107312@gmail.com (X.-H.L.); 2 Department of Obstetrics & Gynecology, E-Da Hospital, E-Da Hospital/I-Shou University, Kaohsiung 82445, Taiwan; E-Mail: jingmei0112@hotmail.com; 3 Department of Nutrition, I-Shou University, Kaohsiung 82445, Taiwan; E-Mail: fen153848@yahoo.com.tw; 4 Department of Biomedical Engineering, I-Shou University, Kaohsiung 82445, Taiwan; E-Mail: smkuo@isu.edu.tw

**Keywords:** *Siegesbeckia orientalis*, RL95-2 endometrial cancer cells, cytotoxicity, apoptosis, ingredient analysis

## Abstract

Endometrial cancer is a common malignancy of the female genital tract. This study demonstrates that *Siegesbeckia orientalis* ethanol extract (SOE) significantly inhibited the proliferation of RL95-2 human endometrial cancer cells. Treating RL95-2 cells with SOE caused cell arrest in the G2/M phase and induced apoptosis of RL95-2 cells by up-regulating Bad, Bak and Bax protein expression and down-regulation of Bcl-2 and Bcl-xL protein expression. Treatment with SOE increased protein expression of caspase-3, -8 and -9 dose-dependently, indicating that apoptosis was through the intrinsic and extrinsic apoptotic pathways. Moreover, SOE was also effective against A549 (lung cancer), Hep G2 (hepatoma), FaDu (pharynx squamous cancer), MDA-MB-231 (breast cancer), and especially on LNCaP (prostate cancer) cell lines. In total, 10 constituents of SOE were identified by Gas chromatography-mass analysis. Caryophyllene oxide and caryophyllene are largely responsible for most cytotoxic activity of SOE against RL95-2 cells. Overall, this study suggests that SOE is a promising anticancer agent for treating endometrial cancer.

## 1. Introduction

Endometrial cancer, the most common gynaecological cancer in developed nations, has an incidence second only to that of cervical cancer [1,2]. The number of cases in Taiwan is increasing steadily [3]. Obesity, diabetes mellitus, endogenous hormones, chronic hyperinsulinemia, late menopause, unopposed estrogen exposure, tamoxifen, the oral contraceptive pill, the reproductive factors parity, and age at birth are considered the primary risk factors [2,4,5,6]. The worldwide increase in obesity and decrease in fertility suggest that the incidence of endometrial cancer will continue to rise, such that endometrial cancer will become a serious public health problem in the future [7]. Currently, the treatment strategies for endometrial cancer are surgery and postoperative adjuvant treatments, such as radiation, chemotherapy and hormonal treatment, especially for advanced endometrial cancer [8,9]. However, the efficiency of these strategies remains limited and their molecular mechanisms still need further investigation. Recently, Chinese herbal medicines have been used as complementary and/or alternative therapies for cancer prevention and treatment [10]. Additionally, the active ingredients of many herbal extracts have been identified and developed into anticancer drugs [11,12].

*Siegesbeckia orientalis* L., which belongs to Asteraceae, has been traditionally taken orally as an anti-inflammation and anti-cancer agent [13], is administered to treat snakebites, cutaneous disorders, rheumatic arthritis [14,15], allergic [16], immune [17], and inflammatory diseases [15]. Wang *et al.* reported that the ethyl acetate and n-butanol extracts of *S. orientalis* markedly inhibited growth of human cervix cancer HeLa cells *in vitro* [18]. However, to the best of our knowledge, the anticancer activity of *S. orientalis* extract on endometrial cancer has not yet been elucidated.

This study evaluates the anticancer potential of *S. orientalis* L. ethanol extract (SOE) for endometrial cancer. Accordingly, the inhibitory effects of SOE on proliferation of endometrial carcinoma RL95-2 cells are investigated. The mechanism of SOE on cell apoptosis is elucidated by analyzing expression of related proteins. Further, the cytotoxicity of SOE on other cancer cell lines is also examined. The chemical constituents of this extract are identified using Gas chromatography-mass (GC-MS) spectrometry. Several bioactive compounds responsible for its anti-proliferative effect on RL95-2 cells are determined as well.

## 2. Results and Discussion

### 2.1. Inhibitory Effect of SOE on RL95-2 Cell Proliferation

**Figure 1:**
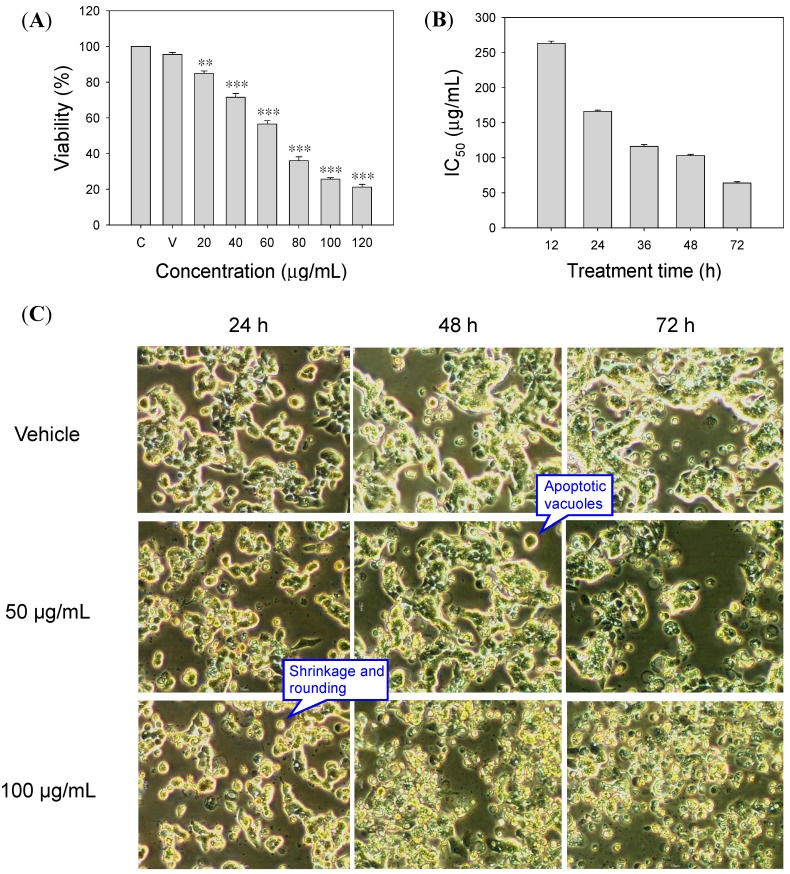
Effects of treatment concentration and duration of SOE on the proliferation of RL95-2 cells. (**A**) Variation in viability under treatment for 72 h with different SOE doses. A significant difference from the Vehicle was indicated as ** *p* < 0.01, or *** *p* < 0.001 by Student’s *t*-test. (**B**) Variation in IC_50_ values under different treatment durations. (**C**) Apoptotic morphological changes of RL95-2 endometrial cancer cells by treatment with SOE at different doses and durations. The RL95-2 cells were cultured in 96-well plates at a density of 1 × 10^4^ cells/well. Cells were treated with medium only (Control, denotes as “C”), DMSO only (Vehicle, denotes as “V”) or media containing 20–120 μg/mL SOE for 12–72 h. Photomicrographs, taken directly from culture plates with a phase-contrast microscope (magnification 20×), show apoptotic cells with shrinkage, rounding and vacuoles.

The inhibitory effect of SOE on the proliferation of RL95-2 cells was examined. Figure 1 shows the inhibitory effect of SOE on the viability of RL95-2 cells after treatment with various SOE concentrations under different incubation durations. Experimental results indicate that SOE inhibited cell viability in dose- and time-dependent manners (Figure 1A,B).

### 2.2. Apoptotic Effects of SOE on RL95-2 Cells

Apoptosis, a programmed cell death, plays a critical role as a protective mechanism against carcinogenesis by eliminating unnecessary or unwanted cells to maintain a healthy balance between cell survival and cell death [19]. The relationship between apoptosis and cancer has been a recent focus. Apoptosis provides many valuable clues about a therapy’s effectiveness, and hence the anticancer effects of many chemotherapeutic agents are *via* apoptosis [20]. Therefore, induction of apoptosis has become a principal focus when developing anticancer therapies.

#### 2.2.1. Cell Morphology

Apoptosis can be characterized by particular morphological changes, such as plasma membrane bleb, cell shrinkage, chromatin condensation and DNA fragmentation [21]. Figure 1C shows the morphological changes of RL95-2 cells after SOE treatment for 24–72 h. Phase-contrast micrographs reveal that SOE induced typical morphological characteristics of apoptosis, including cell shrinkage, apoptotic vacuoles, membrane blebbing and formation of floating cells, in a dose- and time-dependent manner. These changes are in agreement with findings in literature on apoptosis [21,22,23].

#### 2.2.2. Cell Cycle Regulation

**Figure 2:**
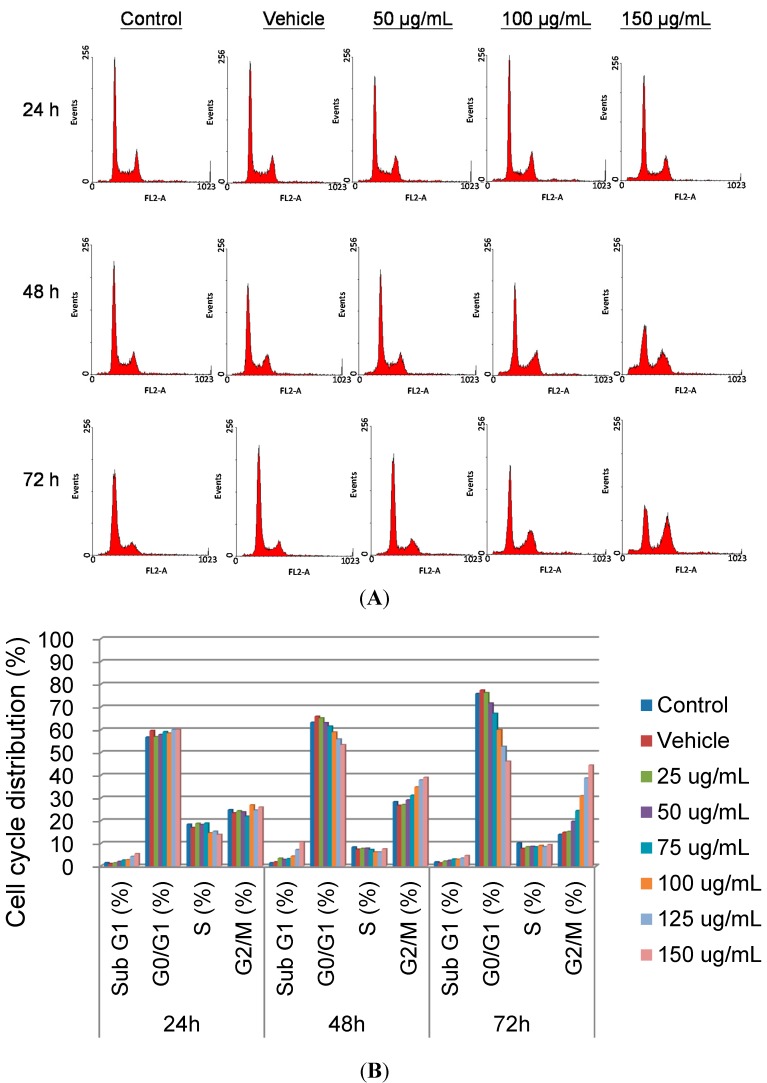
Effect of SOE on cell-cycle progression in RL95-2 cells. (**A**) Cell cycle analysis of RL95-2 cells under treatment with different SOE concentrations for various durations by flow cytometry. The RL95-2 cells (2 × 10^5^ cells/well) were incubated with 0–150 μg/mL of SOE for 24, 48 and 72 h, as indicated in each graph. Cells were suspended in PBS containing 20 μg/mL PI, 0.2 mg/mL RNase A and 0.1% Triton X-100 at 4 °C for 12 h. The stained cells were analyzed by flow cytometry; (**B**) Cell distribution at different phases of cell cycle. The percentage of each phase was analyzed using WinMDI 2.9 software.

To determine whether SOE induced the arrest of cell cycle progression in RL95-2 cells, flow cytometry was applied to quantitate the cell cycle distribution under treatment with different SOE concentrations (0–150 μg/mL) for various durations (24–72 h). The number of cells in the G2/M phase increased significantly, and that in the G_0_/G_1_ phase decreased in SOE-treated cells as the SOE dose increased during 48–72 h treatment (Figure 2). Moreover, this effect also increased over treatment time. This experimental finding implies that SOE could arrest RL95-2 cells at G2/M phase.

**Figure 3:**
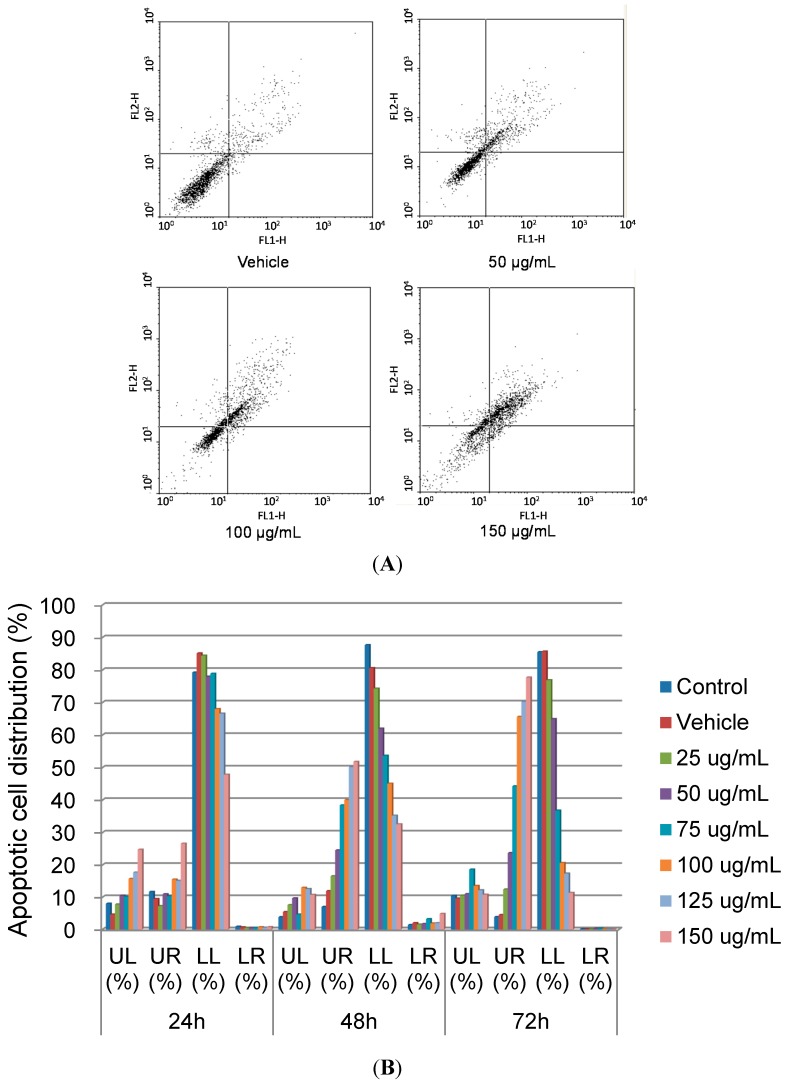
Effect of SOE on RL95-2 cell apoptosis. (**A**) The RL95-2 cells (2 × 10^5^ cells/well) were incubated for 48 h with DMSO only (vehicle, 0.1% v/v) or media containing 50–150 μg/mL SOE. The treated cells were stained with Annexin V-FITC solution and analyzed by flow cytometry. (**B**) Cell distribution under the treatment with 0–150 μg/mL of SOE for 24, 48 and 72 h. Lower left (LL) quadrant: viable cells; lower right (LR) quadrant: early apoptotic cells; upper right (UR) quadrant: late apoptotic cells; upper left (UL) quadrant: necrotic cells.

The Annexin V-FITC apoptosis detection kit was then used to examine the effect of SOE on RL95-2 cell death by flow cytometry. Figure 3 shows that the cell dots were dispersed and shifted to the lower right side, indicating that the cells moved to the early apoptotic stage. When the SOE dose or treatment time increased, the number of cell dots in the upper right side increased, implying that cells progressed gradually to the late apoptotic stage. Conversely, the number of cell dots on the upper left side was not significantly changed, implying that only few cells died via necrosis. These experimental results demonstrate that SOE induced apoptosis of RL95-2 cells.

#### 2.2.3. Pro-Apoptotic and Anti-Apoptotic Proteins Expression

**Figure 4:**
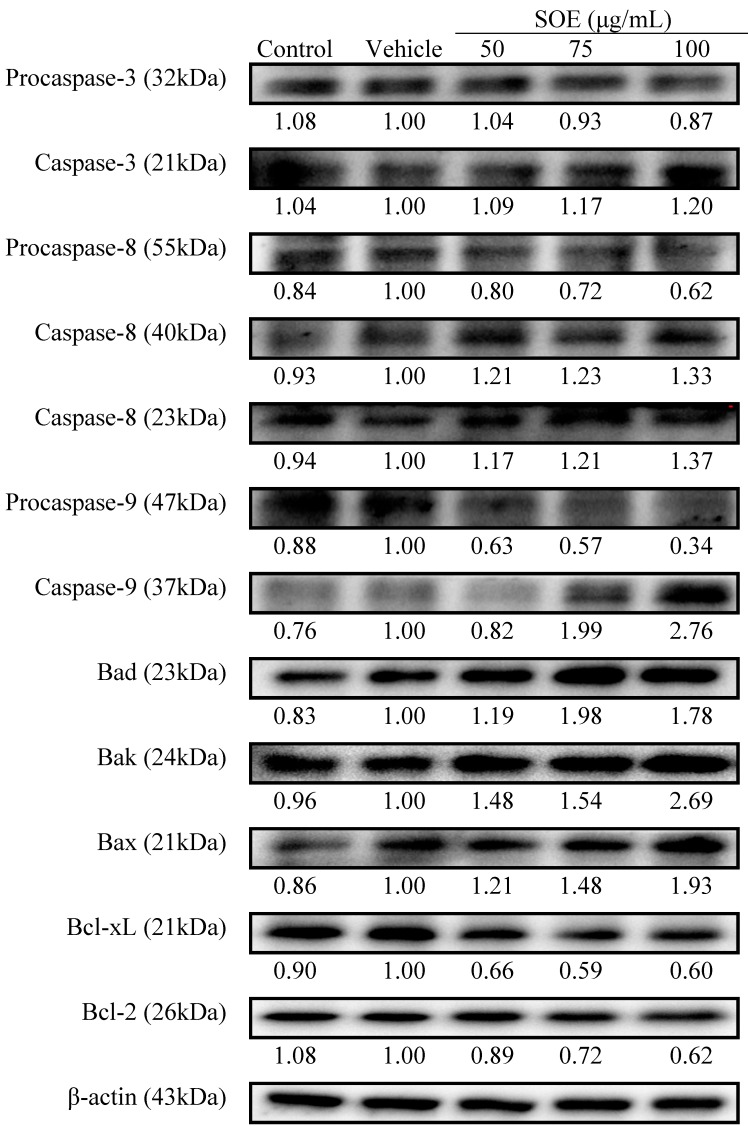
Western blot analysis of apoptosis-related proteins in RL95-2 cells treated with SOE. Caspase-3, -8, -9, Bad, Bak, Bax, Bcl-xL and Bcl-2 proteins in RL95-2 cells after treatment with 0–100 μg/mL SOE for 24 h were detected. Whole-cell lysates were subjected to Western blot assays and β-actin was used as an internal control. The relative density of the proteins was determined by densitometric analysis. The values indicate the density proportion of each protein compared with vehicle.

Apoptosis can occur through two fundamental pathways: (1) the mitochondrial or intrinsic pathway; and (2) the death receptor or extrinsic pathway [24]. Caspase-9 and -8 are the protease indexes for the intrinsic and the extrinsic pathway, respectively. Caspase-3, a well-known downstream adaptor caspase, can be activated by caspase-9 or -8 *via* the intrinsic or extrinsic signaling pathways [20]. To elucidate the molecular effector pathway of SOE-mediated apoptosis, this study determined whether caspases are involved as downstream effectors. As shown in Figure 4, SOE increased cleavage of procaspase-3, -8 and -9, accompanied by an increase in caspase-3, -8 and -9 expression in a dose-dependent manner. This implies that apoptosis was through the intrinsic and extrinsic pathways.

Several cytoplasmic proteins are involved in regulation of apoptosis; particularly members of the Bcl-2 family; one subgroup, including Bcl-2 and Bcl-xL, inhibits apoptosis, while the other, including Bad, Bak and Bax, promotes cell death [20,25]. Since SOE induces the apoptosis of RL95-2 cells, the effect of this extract on cellular proteins that are involved in apoptosis was examined. As the SOE concentration increased, the expressions of Bad, Bak and Bax proteins increased, while those of the Bcl-2 and Bcl-xL proteins decreased (Figure 4). These experimental findings suggest that SOE induced apoptosis of RL95-2 cells.

### 2.3. Cytotoxicity of SOE on Various Cancer Cell Lines

To investigate the potential effect of SOE against other cancers, various cancer cell lines were used to assess the cytotoxicity of SOE and quantified using the MTT assay. Notably, as shown in Table 1, SOE was effective against all these cell lines, and particularly on LNCaP human prostate cancer cells (IC_50_ = 87.2 ± 1.3 μg/mL under 24 h treatment).

**Table 1:** Cytotoxity of SOE on various cancer cell lines ^a^.

Cell Type	Cell Line	IC_50_ (μg/mL) ^b^
Human endometrial carcinoma	RL95-2	163.5 ± 3.3
Human lung carcinoma	A549	179.1 ± 1.8
Human hepatoblastoma	Hep G2	135.4 ± 2.4
Human pharynx squamous cell carcinoma	FaDu	105.0 ± 1.8
Human breast adenocarcinoma	MDA-MB-231	124.3 ± 4.0
Human prostate carcinoma	LNCaP	87.2 ± 1.3

^a^ Treatment time was 24 h; ^b^ Results are expressed as mean ± SD of five experiments.

### 2.4. Cytotoxicity of Main Ingredients of SOE on RL95-2 Cells

**Figure 5:**
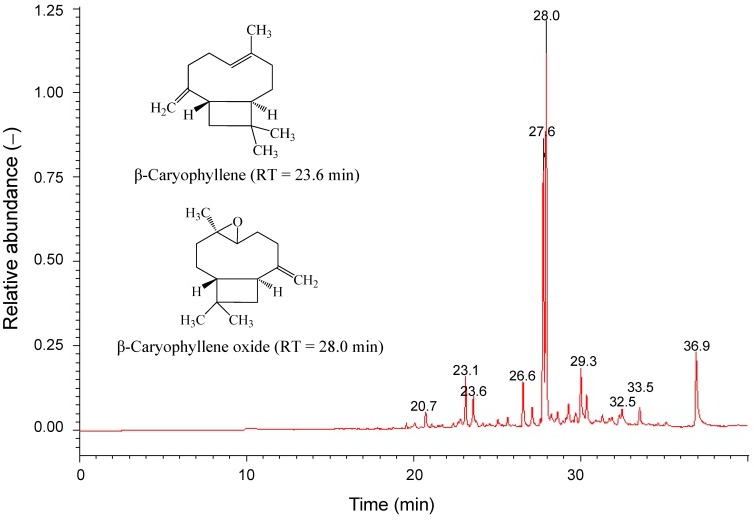
Gas chromatography-mass spectrometry profile of SOE sample.

The GC-MS analytical results show that SOE contains 20 compounds at minimum (Figure 5), of which 10 constituents were identified using mass spectrometry (Table 2). The mass spectra of these compounds were matched with those in the NIST spectral database. The major compounds in SOE were identified as caryophyllene oxide (CPO, 46.9%), [−]-spathulenol (25.7%), hexadecanoic acid ethyl ester (9.6%) and caryophyllene (CP, 3.1%). The CPO, CP, 6,10,14-trimethyl-2-pentadecanone and hexadecanoic acid ethyl ester were confirmed by comparing their mass spectral data with the NIST mass spectral library and commercially available products. The assessment of cytotoxicity of these four compounds on RL95-2 cells for 24 h treatment indicates that only CPO (IC_50_ value = 14.9 ± 0.4 μg/mL (67.7 μM)) and CP (IC_50_ value = 33.2 ± 1.0 μg/mL (162.7 μM)) had significant anti-proliferative activities on RL95-2 cells.

Both CPO and CP are sesquiterpenes isolated mainly from the essential oils of such medicinal plants as *Ocimum basilicum,* *Tagetes minuta* [26], *Toona sinensis* [27], *Hyptis spicigera* and *Lippia multiflora* [28]. Caryophyllene had anti-proliferative ability on many cancer cell lines, including oral, liver, lung, colon, melanoma, leukemia and erythroleukemia [29,30,31]. Notably, CPO inhibited growth and induced apoptosis through suppression of the PI3K/AKT/mTOR/S6K1 pathways and ROS-mediated MAPKs activation in human prostate and breast cancer cells [32,33]. According to Kim *et al.* [34], CPO retarded proliferation, induced apoptosis and abrogated the invasion by suppressing constitutive and inducible STAT3 activation in multiple myeloma, breast and prostate cancer cell lines. Therefore, these two ingredients may be responsible for the cytotoxicity of SOE against human endometrial carcinoma cell line RL95-2.

**Table 2:** Chemical composition of SOE analyzed by GC-MS.

No.	Component	Rt (min) ^a^	Percentage (%) ^b^	R. Match
1	2-Oxabicyclo[2,2,2]octane-6-ol	20.72	1.8	763
2	2-tert-Butyl-1,4-dimethoxy-benzene	23.13	3.8	805
3	Caryophyllene	23.59	3.1	866
4	*cis*-α-Bisabolene	26.57	4.1	860
5	[−]-Spathulenol	27.62	25.7	853
6	Caryophyllene oxide	27.95	46.9	858
7	*cis*-Lanceol	29.28	1.7	719
8	[Z,Z,Z]-9,12,15-Octadecatrienoic acid ethyl ester	32.49	1.2	736
9	6,10,14-Trimethyl-2-pentadecanone	33.53	2.1	790
10	Hexadecanoic acid ethyl ester	36.93	9.6	794

^a^ Retention time (min); ^b^ Relative percentage calculated by integrated peak area.

Although these two compounds have a very slight structural difference, CPO exerted remarkably stronger anti-proliferative activity than CP on RL95-2 cells. Sain *et al.* demonstrated that CPO may have higher cytotoxicity than CP, on human T lymphocyte Jurkat cells and human neuroblastoma IMR-32 cells, from a structural biology *in silico* model based on the degree of stability of the complexes formed between arachidonate 15-lipoxygenase and two caryophyllenes [35]. Additionally, Oh *et al.* reported that CPO had a higher acaricidal activity against house dust mites than CP [36]. These studies revealed that a slight structural difference of CP may significantly affect their bioactivities.

## 3. Experimental Section

### 3.1. Reagents and Materials

The *S. orientalis* L. plant materials were purchased from Yuanshan Company (Kaohsiung, Taiwan). The sample’s original was identified and its nucleotide sequence was deposited in the GenBank database with accession number JN987228 [37]. Dulbecco’s Modified Eagle Medium (DMEM) and fetal bovine serum (FBS) were from Gibco (Grand Island, NY, USA). RNase A was purchased from Gentra Systems Inc. (MN, USA). Annexin V-FITC apoptosis detection kit was obtained from Strong Biotech Co. (Taipei, Taiwan). Procaspase-3, procaspase-8, procaspase-9, Bcl-2, Bcl-xL, Bax, Bad, Bak and β-actin were purchased from BD Biosciences (San Jose, CA, USA). 3-(4,5-Dimethylthiazol-2-yl)-2,5-diphenyltetrazolium bromide (MTT) assay kit and the potential active compounds, caryophyllene, caryophyllene oxide and hexadecanoic acid ethyl ester were from Sigma-Aldrich Chemicals (St. Louis, MO, USA). The compound 6,10,14-trimethyl-2-pentadecanone was from Apollo Scientific Co. (Stockport, Cheshire, UK).

### 3.2. Preparation of S. orientalis Ethanol Extract

The aerial part of *S. orientalis* L. was freeze-dried and ground into powder. The dried powder (9.3 kg) was extracted with 47 L of 95% ethanol by stirring at room temperature for 1 day; this was repeated 3 times. The extracted solutions were collected and filtered through filter paper (Whatman No. 1; Whatman Paper Ltd, Maidstone, Kent, UK). The SOE was obtained by removing solvent with a rotary evaporator and dried it in a freeze-drier. Total dry weight of this extract was 489 g (extraction yield = 5.3%). In cell culture test, the SOE was dissolved in dimethyl sulfoxide (DMSO) and the final DMSO concentration was less than 0.1% in medium.

### 3.3. Cancer Cell Lines and Culture

Human RL95-2 (endometrial cancer), A549 (lung cancer), Hep G2 (hepatoma), FaDu (pharynx squamous cancer), MDA-MB-231 (breast cancer) and LNCaP (prostate cancer) cell lines were purchased from the Bioresource Collection and Research Center (BCRC, Food Industry Research and Development Institute, Hsinchu, Taiwan). The cultivation of RL95-2, A549, Hep G2, FaDu and MDA-MB-231 cell lines were grown in DMEM medium, while LNCaP cells were grown in RPMI 1640 medium. Both of these two media were supplemented with 10% (v/v) FBS, 1% penicillin/streptomycin and 0.02% sodium bicarbonate. These cancer cells were cultivated at 37 °C with 5% CO_2_ and 95% air and in 100% relative humidity.

### 3.4. Determination of Cytotoxicity for Cancer Cells

Cancer cells were cultured in 96-well plates at 1 × 10^4^ cells/well, treated with the indicated concentration of SOE, and cultivated in 100% relative humidity of 5% CO_2_ and 37 °C. After specified cultivation duration, the medium solution was removed. An aliquot of 100 µL culture medium containing 0.5 mg/mL of MTT assay kit was loaded onto the plate. The cells were cultured for 2 h, and then the medium solution was removed. An aliquot of 100 µL DMSO was added and the plate was shaken until its crystals dissolved. The cytotoxicity against cancer cells was determined by measuring the absorbance of the converted dye at a wavelength of 570 nm with an ELISA reader (Model 550, Bio-Rad Laboratories, Hercules, CA, USA). Cytotoxicity of each sample is expressed as IC_50_ value, which is the concentration of test sample that cause 50% inhibition or cell death, and was obtained by plotting the percentage inhibition *versus* SOE concentration.

### 3.5. Flow Cytometry Analysis of Cell Cycle

The effect of SOE on cell cycle distribution was investigated by flow cytometry after staining the cells with propidium iodide (PI). The RL95-2 cells (2 × 10^5^ cells/well) in 24-well plates were treated with various concentrations of SOE and cultured for 24, 48 or 72 h. The treated cells were harvested and washed with phosphate-buffer saline (PBS). Next, 100 μL Trypsin-EDTA solution were added to detach the cells. After centrifugation at 100 *g* for 5 min, the cell pellet was suspended with 70% ethanol and kept at −20 °C for 12 h. Then, the cells were washed with cold PBS and suspended in PBS containing 20 μg/mL PI, 0.2 mg/mL RNase A and 0.1% Triton X-100 at 4 °C for 12 h. The stained cells were then analyzed by flow cytometer (FACSCalibur System, BD Biosciences) and the data were calculated with WinMDI software (Version 2.9, TSRI, La Jolla, CA, USA).

### 3.6. Apoptotic Ratio Analysis

The apoptotic effect of SOE on RL95-2 cells was determined by Annexin V-FITC staining method and measured using flow cytometer. The RL95-2 cells (2 × 10^5^ cells/well) in 24-well plates were treated with various SOE concentrations and cultured for 48 h. The treated cells were harvested and washed with PBS. Next, 100 μL Trypsin-EDTA solution was added to detach the cells. After washing with cold PBS, the cells were centrifuged at 200 *g* for 10 min. The cell pellet was suspended in 100 μL of Annexin V-FITC staining-solution and incubated for 20–30 min at 25 °C. The cells were then analyzed by flow cytometry.

### 3.7. Preparation of Whole-Cell Lysates

The composition of cell lysis buffer (modified RIPA buffer) was 150 mM NaCl, 50 mM Tris, 0.5% sodium deoxycholate, 1% tert-octylphenoxy poly(oxyethylene)ethanol (IGEPAL), 20 μL of 0.1 M ethylene glycol tetraacetic acid (EGTA), 2 μL of 0.25 M sodium vanadat, 10 μL of 0.1 M phenylmethylsulfonyl fluoride (PMSF), 2 μL of 5 mg/mL leupeptin, 2 μL of 5 mg/mL aprotinin, and 2 μL of 0.5 M ethylene-diaminetetraacetic acid (EDTA). The cultivated cells were rinsed with iced-cold PBS and lysed with 100 μL cold modified RIPA buffer for 5 min. Supernatants were collected by centrifugation at 10,000 *g* for 5 min at 4 °C, and were used as the whole-cell lysates.

### 3.8. Western Blot Analysis

To analyze the proteins of procaspase-3, procaspase-8, procaspase-9, Bcl-family, 1 × 10^5^ cells were seeded into 6-cm culture dishes with or without SOE, and were incubated for 24 h. The medium was removed and cells were washed several times with PBS (0.01 M, pH 7.2). Whole-cell lysates were prepared using the procedures described earlier. The harvested protein concentration was measured using a protein assay kit (Bio-Rad). The same amounts of proteins from each extract were applied to 10% sodium dodecyl sulfate polyacrylamide gel electrophoresis (SDS-PAGE). Proteins were transferred onto a nitrocellulose membrane (Immunobilin P; Millipore, Billerica, MA, USA), and were then blocked by 10% skim milk in water for 1 h. After washing three times with PBS containing 0.1% Tween-20, the specific primary antibodies with a suitable dilution were added. Following overnight incubation at 4 °C, the primary antibodies were washed away and secondary antibodies were added for 1 h incubation at room temperature. The protein levels were determined by using Enhanced Chemiluminescence (ECL) Plus Western blotting detection reagents (Amersham Bioscience, Uppsala, Sweden) to develop the signal of the membrane. Densitometric analyses were conducted using the Quantity One^®^ software (Bio-Rad).

### 3.9. Gas Chromatography-Mass Spectrometry

The GC-MS analysis was performed using Varian 450-GC and 240-MS system (Varian, Salt Lake City, UT, USA) with the electron impact mode (70 eV) injector, and a Varian data system. The GC column was VF-5ms capillary column (30 m × 0.25 mm, film thickness 0.25 μm, FactorFour^TM^, Varian, Salt Lake City, UT, USA). Injector and detector temperatures were set at 250 °C and 290 °C, respectively. Oven temperature was kept at 50 °C for 5 min, then raised to 120 °C by a rate of 5 °C/min, kept at 120 °C for 8 min, then raised to 300 °C by a rate of 10 °C/min. The carrier gas was helium at a flow rate of 1 mL/min. Diluted samples of 1.0 μL was injected under the splitless mode. The percentages of the ingredients were calculated by the area normalization method. The components were identified by comparison of their mass spectra with the NIST MS 2.0 database (Gaithersburg, MD, USA).

### 3.10. Statistical Analysis

All experiments were carried out for three to five independent replicates. The experimental data were analyzed by using Microsoft Excel software (Microsoft Software Inc., Redmond, WA, USA). The data are expressed in terms of mean and standard deviation, and the statistical differences were analyzed by Student’s *t*-test (* *p* < 0.05, ** *p* < 0.01, *** *p* < 0.001).

## 4. Conclusions

This study demonstrates that SOE had significant cytotoxicity directly towards RL95-2 endometrial cancer cells. Under SOE treatment, the cell morphology change, cell cycle regulation, apoptosis by Annexin V-FITC detection, and expressions of pro-apoptotic and anti-apoptotic proteins expression of RL95-2 cells were investigated. Notably, SOE induced apoptosis of RL95-2 cells via both intrinsic and extrinsic signaling pathways. Further, CPO and CP were responsible for most of the cytotoxicity of SOE. Taken together, these experimental findings suggest that SOE is a potential source for both the prevention and treatment of endometrial cancer. Further studies are required to identify the bioactive components and their mechanisms of action.
